# Using an online calculator to describe excess mortality in the Philippines during the COVID-19 pandemic

**DOI:** 10.5365/wpsar.2023.14.1.984

**Published:** 2023-03-22

**Authors:** Julius R Migriño, Ma Rosario Bernardo-Lazaro

**Affiliations:** a San Beda University College of Medicine, Manila, Philippines.; b Ateneo de Manila University School of Medicine and Public Health, Pasig, Philippines.

## Abstract

**Objective:**

Excess mortality is an indicator of the impact of the coronavirus disease (COVID-19) pandemic. This study aims to describe excess mortality in the Philippines from January 2020 to December 2021 using an online all-cause mortality and excess mortality calculator.

**Methods:**

All-cause mortality data sets from 2015 to 2021 from the Philippine Statistics Authority were obtained and analysed using the World Health Organization Western Pacific Regional Office All-Cause Mortality Calculator. Expected mortality, excess mortality and P-scores were obtained using two models, 5-year averages and negative binomial regression, for total deaths and by administrative region.

**Results:**

Reported national all-cause mortality exceeded the expected mortality in August 2020 and from January to November 2021, peaking in September 2021 at 104 per 100 000. Total excess mortality using negative binomial regression was −13 900 deaths in 2020 and 212 000 deaths in 2021, peaking in September 2021. P-scores were −2% in 2020 and 33% in 2021, again peaking in September 2021 at 114%. Reported COVID-19 deaths accounted for 20% of excess deaths in 2021. In 2020, consistently high P-scores were recorded in the National Capital Region from July to September and in the Bangsamoro Autonomous Region in Muslim Mindanao from June to July. In 2021, most regions recorded high P-scores from June to October.

**Discussion:**

Tracking excess mortality using a robust, accessible and standardized online tool provided a comprehensive assessment of the direct and indirect impacts of the COVID-19 pandemic in the Philippines. Furthermore, analysis by administrative region highlighted the key regions disproportionately affected by the pandemic, information that may not have been fully captured from routine COVID-19 surveillance.

The coronavirus disease (COVID-19) pandemic has been ongoing since March 2020 and, as of early January 2023, there have been more than 666 million reported cases and more than 6.7 million reported deaths globally. (*1*) The numbers of daily or weekly COVID-19 cases and deaths have been used to assess the impact of the pandemic. However, while data on COVID-19-related deaths have been widely reported, the quality, accuracy and timeliness of mortality data can be influenced by country-specific factors such as COVID-19 testing capacity, population and per capita income, (*2*, *3*) and are often underreported or delayed especially in low-income countries. (*4*, *5*) Therefore, reported COVID-19 mortality data may not reflect the full impact of the pandemic. An assessment by the World Health Organization (WHO) of 133 countries in 2020 found that almost 40% of the world’s deaths were not registered. (*6*)

One method to standardize estimates of COVID-19 deaths is through measurement of excess mortality, defined as “the increase of all-cause mortality over the mortality expected based on historic trends.” (*7*) P-score is an associated index of excess mortality and represents the percentage of excess deaths relative to the expected deaths. (*8*) In a 2020 study, excess mortality and P-scores were reported for most countries, particularly those in Central and South America, (*8*) with global estimates of excess mortality for 2021 of 18.2 million people, more than three times the reported global COVID-19 deaths. (*7*) Excess mortality and P-scores provide more realistic estimates of the true mortality during the COVID-19 pandemic, which includes estimates of underreported COVID-19 deaths as well as indirect deaths, that is, those from other diseases. (*8*, *9*)

The Philippines is an archipelagic lower middle-income country divided into 13 administrative regions (**Map 1**). Country data on mortality are available from both the Philippine Statistics Authority (PSA) and the Department of Health (DOH). The PSA data are obtained from death certificates, whereas the DOH data are obtained from mortality reports sent to the DOH Epidemiology Bureau. (*10*) There have been several reports on excess mortality in the Philippines, with low and negative excess mortality reported compared to other countries in the region; (*7*, *11*) one study also reported that the excess mortality rate in the Philippines was almost 3.5 times the recorded number of COVID-19 deaths. (*7*)

The World Health Organization Western Pacific Regional Office All-Cause Mortality Calculator (“ACM Calculator”) is an open-source online tool developed to calculate expected all-cause mortality, excess mortality and P-scores from mortality data. All-cause mortality can also be calculated by age, sex and administrative state or region if the disaggregated mortality data are indexed in the calculator. The results can then be displayed using tables and graphs. The ACM Calculator estimates excess mortality and P-scores using two approaches: historical 5-year averages (5YA) and a non-parametric negative binomial regression (NBR) model. (*12*)

The aim of this study is to describe excess mortality in the Philippines at national and regional levels during the COVID-19 pandemic (2020 and 2021) using data generated by the ACM Calculator.

## Methods

Mortality data from publicly available PSA reports (*13*) from 2015 to 2021 were obtained by year, month and region. These data were encoded into a blank template provided by the ACM Calculator web site and used to generate the following statistics in the Calculator. (*14*, *15*)

*Expected all-cause mortality and 95% confidence intervals using 5YA.* This statistic takes the monthly average and 95% confidence intervals of the reported mortality using data from 2015 to 2019.
*Expected all-cause mortality and 95% prediction intervals using NBR.* This statistic uses an NBR approach to estimate deaths for 2020 and 2021 using data from 2015 to 2019. This technique is preferred since it allows for overdispersion and can also account for low or zero counts. The mean parameter (𝜆𝑡) for the counts is modelled as
  **log** 𝜆𝑡 = 𝑐(𝑡) + 𝑡𝑟𝑒𝑛𝑑(𝑡) + 𝑋𝑡𝛽

where *c(t)* is the annual cycle in all-cause mortality, modelled as a piecewise cyclic cubic spline function, *trend(t)* is the non-cyclic cubic spline function of all-cause mortality over time, and *Xt* is for arbitrary time-varying covariates.

*Excess mortality.* Excess mortality was calculated using the formula
  excess mortality = reported mortality – expected mortality

and values were calculated per region and per month for 2020 and 2021. Excess mortality counts were computed using both 5YA and NBR expected mortality.

*P-scores of excess mortality*. P-score was calculated using the formula
  P-score = excess mortality/expected mortality x 100

and is expressed as percentages. These values were calculated per region and per month for 2020 and 2021 and were also computed using both 5YA and NBR expected mortality.

Total excess mortality and P-scores were calculated using both 5YA and NBR expected mortality. However, only NBR was used to calculate excess mortality and P-scores per administrative region due to its increased accuracy and adoption by WHO.

Reported COVID-19 deaths per month for 2020 and 2021 were also extracted from the WHO coronavirus (COVID-19) dashboard. (*16*) The ratio of COVID-19 deaths to excess deaths was calculated using the formula

Ratio = COVID-19 deaths/excess mortality x 100

All raw data on reported mortality as well as calculated statistics were tabulated. Time-series line graphs for reported mortality, expected mortality and P-scores were generated, and box plots for excess mortality were created. Data entry, cleaning and processing were completed in Microsoft Excel.

## Results

**Fig. 1:**
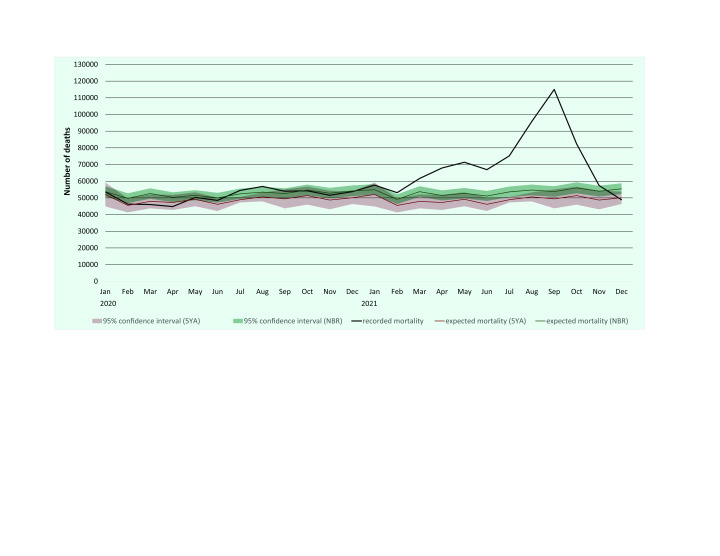
Number of reported deaths and expected deaths calculated using the negative binomial regression and 5-year average methods, the Philippines, 2020 and 2021

In 2020, reported mortality in the Philippines peaked during August at 52 per 100 000 population, with the lowest mortality rate reported in April at 41 per 100 000. In 2021, the peak occurred in September at 104 per 100 000, with the lowest rate observed for December at 44 per 100 000 (**Fig. 1**). The reported mortality for the Philippines exceeded the upper bound of the expected mortality in August 2020 and from January to November 2021, while mortality was lower than expected in March and April 2020 and in December 2021 (**Fig. 1**).

[**insert** Figure 1]

The total excess mortality using the NBR method for the Philippines was −13 900 deaths, or −13 deaths per 100 000 population for 2020, and 212 900 deaths or 193 deaths per 100 000 population for 2021. P-scores were −2% for 2020 and 33% for 2021 (**Table 1**). The highest excess mortality (56 per 100 000 population) and P-score (114%) were recorded in September 2021. The calculated excess mortality was lower using NBR compared to 5YA across all time points (**Table 1**).

**Table 1:** Reported number of deaths and number, rate and P-score of excess deaths calculated using the negative binomial regression and 5-year average methods, the Philippines, 2020 and 2021

Year and month	Reported mortality^b^	Excess deaths using negative binomial regression		Excess deaths using 5-year average method	
	Number (rate per 100 000 population)	Number (rate per 100 000 population)^b^	P-score (%)^c^	Number (rate per 100 000 population)^b^	P-score (%)^c^
**2020 total^a^**	**613 900 (563)**	**-13 900 (−13)**	**-2%**	**27 200 (25)**	**5%**
January	53 500 (49)	−200 (0)	0%	1500 (1)	3%
February	46 300 (42)	−3500 (−3)	−7%	800 (1)	2%
March	46 000 (42)	−6600 (−6)	−12%	−1900 (−2)	−4%
April	44 800 (41)	−5500 (−5)	−11%	−2500 (−2)	−5%
May	50 300 (46)	−1300 (−1)	−3%	1100 (1)	2%
June	48 500 (44)	−1500 (−1)	−3%	2400 (2)	5%
July	54 400 (50)	2000 (2)	4%	5600 (5)	11%
August	56 800 (52)	3400 (3)	6%	6300 (6)	12%
September	54 000 (49)	1400 (1)	3%	4600 (4)	9%
October	54 200 (50)	−500 (0)	−1%	2900 (3)	6%
November	51 600 (47)	−1200 (−1)	−2%	2900 (3)	6%
December	53 700 (49)	−400 (0)	−1%	3700 (3)	7%
**2021 total^a^**	**853 100 (774)**	**212 900 (193)**	**33%**	**266 400 (242)**	**45%**
January	57 600 (52)	2700 (2)	5%	5600 (5)	11%
February	53 100 (48)	4000 (4)	8%	7700 (7)	17%
March	61 600 (56)	7900 (7)	15%	13 700 (12)	29%
April	67 900 (62)	16 500 (15)	32%	20 700 (19)	44%
May	71 400 (65)	18 600 (17)	35%	22 300 (20)	45%
June	66 900 (61)	15 900 (14)	31%	20 800 (19)	45%
July	75 200 (68)	21 600 (20)	40%	26 300 (24)	54%
August	95 700 (87)	41 100 (37)	75%	45 200 (41)	89%
September	115 000 (104)	61 200 (56)	114%	65 600 (60)	133%
October	82 400 (75)	26 500 (24)	47%	31 100 (28)	61%
November	57 400 (52)	3400 (3)	6%	8700 (8)	18%
December	48 900 (44)	−6500 (−6)	−12%	−1100 (−1)	−2%

^a^ Cumulative counts of excess mortality per year may not reflect the sum of values shown due to rounding.

^b^ Mortality counts 100 and above were rounded to the nearest 100; mortality counts below 100 were rounded to the nearest 10; rates were rounded to the nearest integer.

^c^ P-scores were rounded to the nearest integer.

**Fig. 2:**
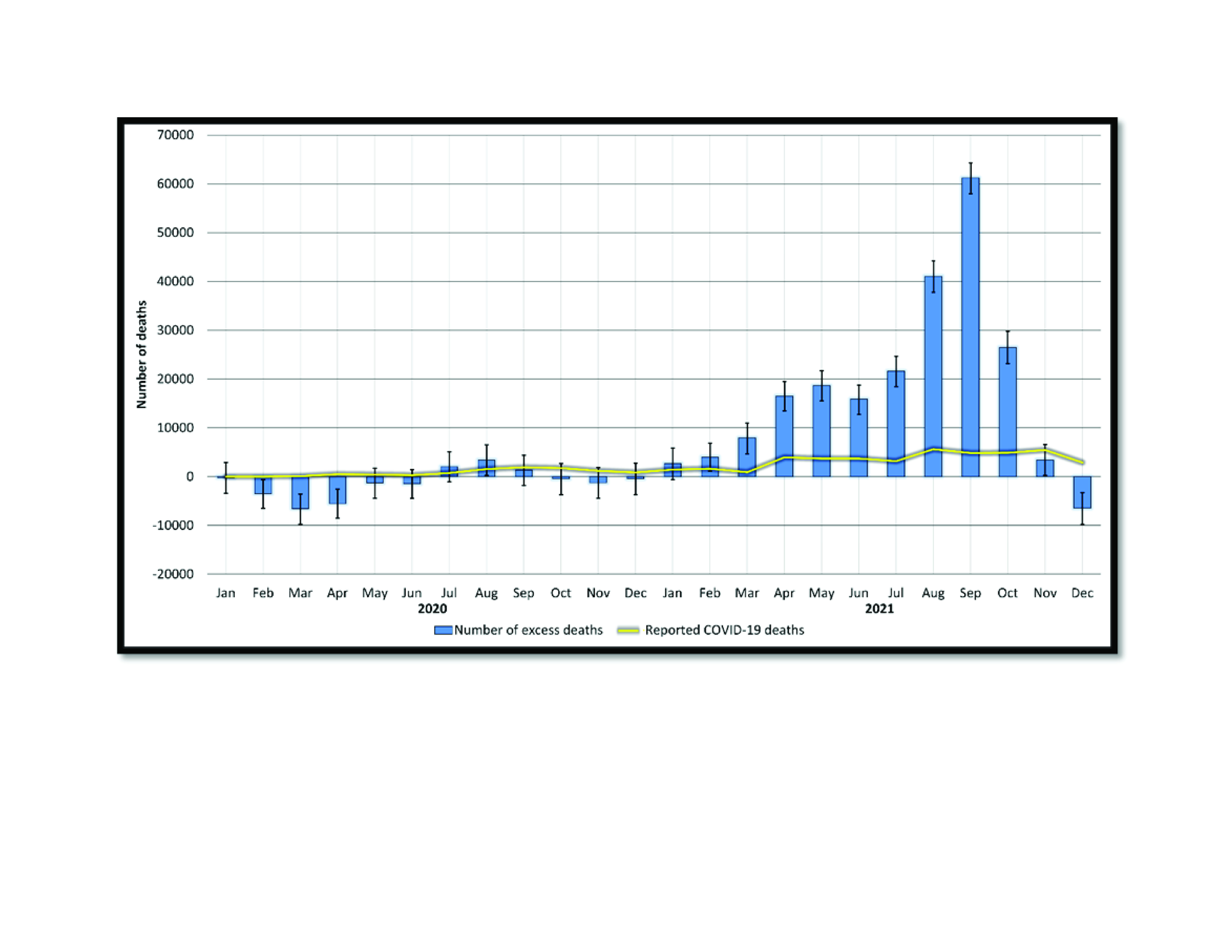
Reported COVID-19 deaths and number of excess deaths calculated using the negative binomial regression method, the Philippines, 2020 and 2021

The ratio of reported COVID-19 deaths to calculated excess mortality was −66% in 2020 and 20% in 2021, the latter suggesting that reported COVID-19 deaths in the country only accounted for about 20% of excess mortality in 2021. Monthly ratios ranged from −360% in October to 142% in September of 2020 (interquartile range = 56%) and from −44% in December to 159% in November of 2021 (interquartile range = 15%) (**Fig. 2**).

[**insert** Figure 2]

In the analysis by administrative region, only the National Capital Region (NCR) reported positive excess mortality of 14 100 deaths or 105 per 100 000 in 2020, while all administrative regions reported positive excess mortality in 2021 (**Table 2**). Overall, for both years, NCR had the highest excess mortality, with Region IV-A and Region III ranking second and third, respectively. The regions with the highest excess mortality rates in 2021 were Regions I, III and IV-A, while Bangsamoro Autonomous Region in Muslim Mindanao (BARMM) had the lowest excess mortality (**Table 2**).

**Table 2:** Number of excess deaths calculated using the negative binomial regression method by administrative region, the Philippines, 2020 and 2021

Region	Number of excess deaths (rate per 100 000 population) by year^a^		
	2020	2021	2020 and 2021^b^
NCR	14 100 (105)	22 500 (161)	36 600
Region I	−1800 (−33)	16 200 (304)	14 400
Region II	−3200 (−86)	8900 (240)	5700
Region III	−6600 (−53)	33 800 (270)	27 200
Region IV-A	−6200 (−39)	39 500 (241)	33 200
Region IV-B	−1900 (−58)	4800 (148)	2900
Region V	−5200 (−86)	9400 (152)	4200
Region VI	−4100 (−52)	15 800 (198)	11 600
Region VII	−900 (−11)	15 500 (192)	14 600
Region VIII	−2700 (−58)	7200 (150)	4600
Region IX	−2100 (−55)	6900 (182)	4800
Region X	−2500 (−49)	6300 (124)	3800
Region XI	−1400 (−27)	9000 (167)	7600
Region XII	−1700 (−40)	8700 (174)	7000
Region XIII	−1900 (−69)	4200 (152)	2300
BARMM	−90 (−2)	1300 (30)	1200
CAR	−1200 (−65)	3400 (186)	2200

BARMM: Bangsamoro Autonomous Region in Muslim Mindanao; CAR: Cordillera Administrative Region; NCR: National Capital Region.

^a^ Mortality counts 100 and above were rounded to the nearest 100; mortality counts below 100 were rounded to the nearest 10; rates were rounded to the nearest integer.

^b^ Totals of excess mortality for 2020 and 2021 may not reflect the sum of values shown due to rounding.

**Fig. 3:**
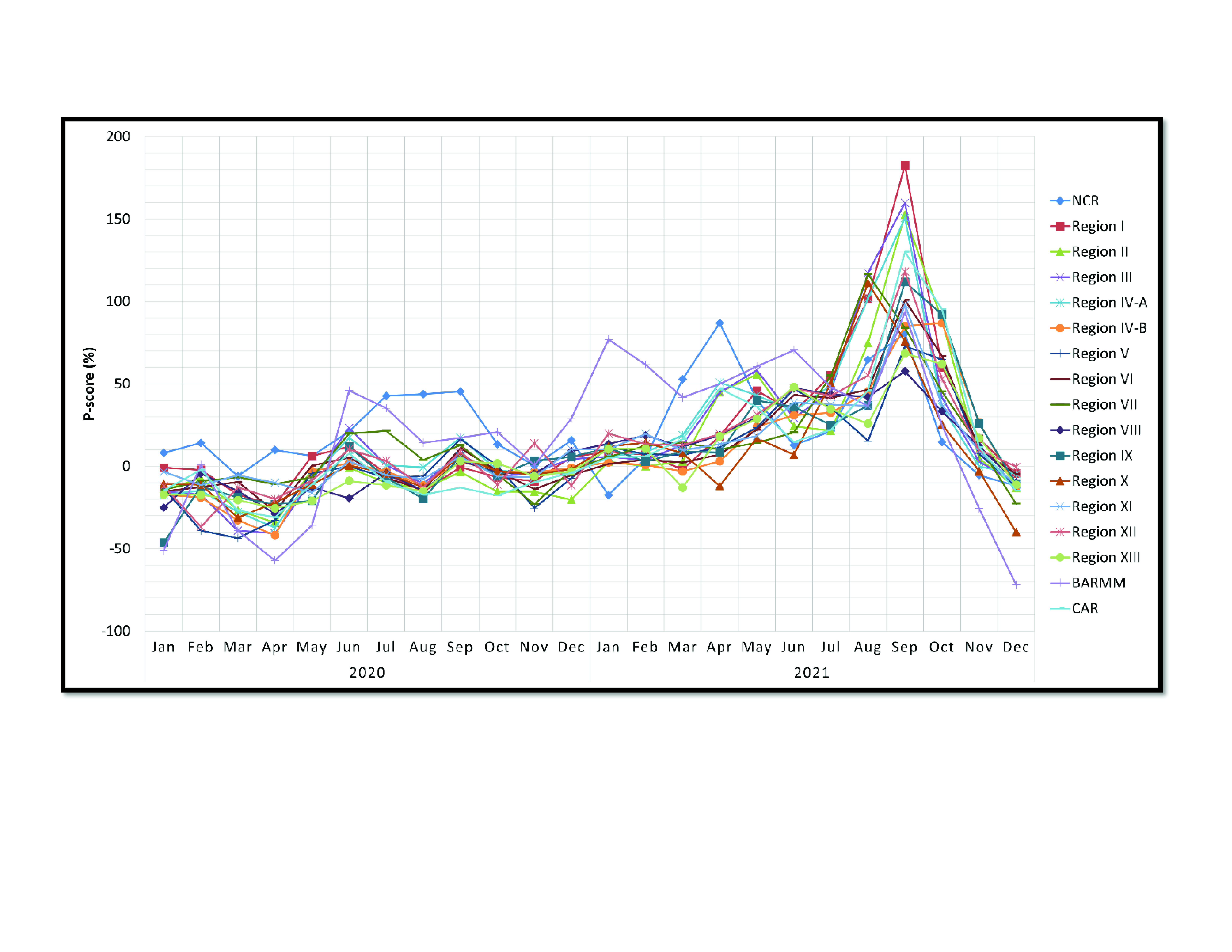
Number of excess deaths calculated using the negative binomial regression method by administrative region, the Philippines, 2020 and 2021

When assessing P-scores per region per month for 2020 and 2021 (**Fig. 3**; **Table 3**), the top three highest monthly P-scores occurred in September 2021 from Region I at 183%, Region III at 160% and Region II at 153%. Up until June 2020, most regions had negative P-scores, whereas in June 2020 only three regions had negative P-scores. NCR had high P-scores (that is, greater than the 75th percentile) from July to September 2020, while BARMM had high P-scores in June and July. In 2021, there were high P-scores for most regions from June to October, while NCR had high P-scores from March to May and from August to September. BARMM consistently had high P-scores from January to September 2021.

[**insert** Figure 3]

**Table 3:** P-scores^a^ calculated using the negative binomial regression method by administrative region and month, the Philippines, 2020 and 2021

P-score (%), 2020												
Region	Jan	Feb	Mar	Apr	May	Jun	Jul	Aug	Sep	Oct	Nov	Dec
NCR	8	14	−6	10	6	21	43	44	45	13	0	16
Region I	−1	−2	−17	−24	6	12	−5	−15	−1	−7	−9	6
Region II	−15	−7	−27	−34	−2	−1	−10	−14	−3	−15	−15	−20
Region III	−15	−17	−39	−41	−8	23	1	−11	8	−7	−3	4
Region IV-A	−17	−15	−28	−37	−7	17	1	−1	17	−3	−4	−4
Region IV-B	−17	−19	−33	−42	−3	2	−3	−12	3	−3	−4	−1
Region V	−13	−39	−44	−33	−4	0	−7	−6	16	0	−25	−7
Region VI	−15	−13	−10	−28	0	5	−7	−15	11	−3	−14	−6
Region VII	−14	−9	−7	−11	−7	20	21	4	13	−5	−23	−2
Region VIII	−25	−4	−15	−29	−13	−19	−4	−15	3	−4	−5	9
Region IX	−46	−12	−19	−23	−21	12	−7	−20	6	−6	3	6
Region X	−11	−11	−31	−22	−11	0	−4	−13	6	−3	−5	−2
Region XI	−3	−11	−6	−10	−16	3	−5	−8	6	−6	−7	10
Region XII	−12	−37	−13	−20	−9	10	3	−13	9	−11	14	−12
Region XIII	−17	−17	−21	−25	−21	−9	−11	−15	3	2	−6	−3
BARMM	−51	1	−39	−57	−36	46	35	14	17	21	1	29
CAR	−15	−2	−27	−31	−11	8	−9	−17	−13	−18	−10	−4
**P-score (%), 2021**												
**Region**	**Jan**	**Feb**	**Mar**	**Apr**	**May**	**Jun**	**Jul**	**Aug**	**Sep**	**Oct**	**Nov**	**Dec**
NCR	−17	5	53	87	40	13	21	65	80	15	−5	−12
Region I	9	7	−2	18	46	33	55	102	183	60	17	−4
Region II	3	0	3	45	56	24	22	75	153	88	5	−13
Region III	6	4	13	45	58	29	45	117	160	42	3	−11
Region IV-A	6	8	19	51	43	33	49	102	151	36	0	−5
Region IV-B	2	1	−3	3	24	31	32	45	85	87	27	−12
Region V	12	7	7	12	23	47	35	15	73	65	8	−12
Region VI	1	4	2	7	22	43	42	46	101	67	10	−5
Region VII	6	12	14	10	14	20	55	117	84	45	12	−23
Region VIII	14	19	12	19	30	47	44	42	58	33	12	−10
Region IX	11	3	9	8	40	36	25	37	112	92	26	−11
Region X	12	14	8	−12	17	7	50	111	76	25	−3	−40
Region XI	10	20	10	13	18	39	37	37	98	37	9	−11
Region XII	20	13	13	20	31	47	43	55	118	53	10	0
Region XIII	10	11	−13	18	29	48	35	26	68	62	17	−11
BARMM	77	62	42	50	61	70	48	37	93	25	−26	−72
CAR	5	6	17	47	36	15	22	46	130	95	12	−15

BARMM: Bangsamoro Autonomous Region in Muslim Mindanao; CAR: Cordillera Administrative Region; NCR: National Capital Region.

^a^ P-scores were rounded to the nearest integer.

## Discussion

By using the ACM Calculator, we showed that the reported all-cause mortality in the Philippines exceeded expectations in July and August 2020, which coincided with the peak of the country’s second wave (*17*) in August 2020. Most months in 2021 recorded a higher mortality rate than expected, which peaked in September. This coincided with the spread of the Delta variant across the Western Pacific Region and throughout the country. (*17*)

As there is a lack of published studies on excess mortality by region for the Philippines, we also calculated excess mortality and P-scores by administrative region. Unlike the results for the Philippines overall, most regions had negative P-scores during July and August 2020. However, the high P-scores in NCR, Region VII and BARMM contributed significantly to the total all-cause mortality recorded during those months. BARMM and NCR recorded positive excess mortality and high P-scores consistently from the start of the pandemic despite having highly different local government structures, population demographics, population density and distribution, and even geospatial characteristics. During 2021, most regions recorded positive excess mortality and P-scores for most months, and all contributed to the higher-than-expected national all-cause mortality rate reported in 2021. This provides further evidence of the country-wide spread of COVID-19 during 2021.

Overcrowding was identified as a factor affecting excess mortality in Chile. (*18*) In this study, regions with the highest population density (that is, NCR, Region III and Region V-A) (*19*) also had the highest total excess mortality from 2020 to 2021. A local study showed a strong positive correlation (r = 0.92) between COVID-19 deaths and regional population density, as well as between the number of intensive care unit beds and doctors absent due to being in quarantine (r = 0.92 and 0.85, respectively). (*20*) Regions III and IV-A border NCR with many workers regularly travelling to NCR from these regions, suggesting labour mobility may have also played a role in excess deaths. However, the above-mentioned local study found low correlation between mobility and COVID-19 deaths. (*20*) Aron et al. recommended supplementing region-based disaggregation analysis with analyses by age, sex and socioeconomic categories (such as inequality and urban density) to reveal “the effectiveness of different types of policy.” (*21*) Nonetheless, an analysis disaggregated by region could contribute to the assessment of the impact of the COVID-19 pandemic, particularly by identifying specific areas that are disproportionately affected.

While this study showed that the patterns of all-cause mortality and excess mortality were similar to reported COVID-19 deaths in the Philippines, these reported COVID-19 deaths only accounted for 20% of the excess mortality in 2021. A recent global study estimated this proportion at 29% for the Philippines from 2020 to 2021, compared to around 85% in high-income countries such as Belgium and Sweden. (*9*) The Commission on Population and Development in the Philippines also acknowledged that COVID-19 was a major contributor to excess mortality in 2021. (*22*) Discrepancies in excess deaths versus reported COVID-19 deaths suggest that there may be: (1) underreporting of actual COVID-19 deaths; (2) a large cohort of deaths indirectly caused by the pandemic that are not COVID-19 deaths; or (3) a combination of both. (*23*-*27*)

Delays in reporting contribute to underreporting of COVID-19 deaths, as can the varying quality, intensity and timing of testing and location of death. Early in the pandemic, data quality was recognized as a possible factor in underestimating COVID-19 deaths, especially in developing countries. (*3*) In some states in the United States of America, increases in excess deaths corresponded to increases in testing intensity. (*28*) In Italy, COVID-19 mortality data did not include deaths at home or in care facilities where COVID-19 testing was not routinely carried out. (*27*) The Philippines DOH released several advisories which acknowledged delays in reporting of COVID-19 mortality data, citing logistical delays from local government units and health-care providers as well as technical issues with the information system as possible reasons for delayed reporting. (*29*, *30*) Often considered the most reliable epidemic indicator internationally, reporting of daily deaths may be unreliable and may peak at times that appear contradictory to patterns of confirmed cases. (*31*)

Indirect deaths caused by the pandemic also contribute to the excess mortality counts, (*11*, *32*) but the exact proportion of indirect deaths is difficult to ascertain, varying by country, state or even locality. Based on our study, indirect deaths appear to be a significant contributor to excess mortality, possibly responsible for as much as 80% of the excess deaths in 2021. In one study in the United States of America, drug overdoses, homicides, suicides and unintentional injuries may have contributed to non-COVID-19 excess deaths in 2020, (*25*) while a study in Latvia documented varying noncommunicable diseases contributing to excess mortality, such as circulatory diseases, neoplasms, diabetes mellitus and chronic lower respiratory diseases. (*33*) Data from the Philippines on non-COVID-19 causes of death in 2021 compared to 2020 showed increases in deaths due to ischaemic heart disease (30%), cerebrovascular disease (15%), diabetes mellitus (21%), hypertensive disease (32%) and malnutrition (47%). (*22*) Cancer-related deaths decreased by 10%, but this was partly attributed to “COVID[-19] cases [crowding] out actual and undiagnosed cancer patients.” (*22*)

Excess mortality is often calculated using two general models: historical (for example, 5-year) averages and NBR. NBR models can be used for data with low or zero counts, and can account for overdispersion, seasonal fluctuations within a given year, secular trends in data, reporting delays and other time-sensitive covariates, such as internal and external movement of populations or periods with low reporting activities (for example, holidays). (*7*, *12*, *14*) Although we used both models to calculate total excess mortality, our analysis focused on the NBR model for two reasons: (1) the accuracy of the NBR model in the ACM Calculator was validated in its documentation; (*14*) and  (2) WHO recently released a document detailing the use of regression models in estimating excess mortality data. (*15*)

The cumulative 2020–2021 excess mortality estimates from our study using the NBR estimate  (199 000) was closer to the estimate (184 000) from a 2022 global study (*9*) from the same time period which used Poisson modelling and a recent WHO-modelled estimate  (185 300) as reported in May 2022. (*24*) Our result using the 5YA model (293 600) was closer to the projected excess deaths (254 897) from a local presentation which also used historical averages and mid-2021 data. (*32*) Variations in study findings are often influenced by the completeness and reliability of the all-cause mortality data used as well as backward revisions of preliminary data. (*7*) Although the trend of excess deaths from both methods used in this study were consistent, the total number of excess deaths differed, suggesting that analysis of excess mortality data should take into account the method used to calculate the excess deaths.

There were several limitations to this study. Mortality reporting systems do not cover all deaths, especially in low-resource settings, with civil registration of deaths noted to be as low as 20% in some low- and middle-income countries. (*1*) Additionally, mortality data are often preliminary which suggests that the earlier data may be more incomplete. (*7*) The ACM Calculator assumes that reported counts are the actual values and that reports are complete and accurate, but it does not currently account for reporting delays. This may explain the overestimation of our data compared to studies from older data sets. Therefore, the results of the ACM Calculator should be interpreted with caution, particularly when there are timeliness issues and reporting delays. (*12*, *14*) Second, our data set did not contain disaggregated data on age, sex and other factors associated with excess mortality, which limited our analysis to administrative regions. Lastly, we were not able to account for regional variations in testing and reporting accuracy and capacity that may have influenced the data set.

Analysing excess mortality provided a more comprehensive picture of the direct and indirect impacts of the COVID-19 pandemic in the Philippines. While the pattern of excess mortality was similar to reported COVID-19 deaths, the reported COVID-19 deaths only accounted for a small proportion of excess deaths. We therefore recommend incorporating excess mortality analysis during surveillance of similar events such as outbreaks and pandemics. Our analysis by administrative region highlighted the key regions disproportionately affected by the pandemic, which is information that may not have been fully captured from national COVID-19 surveillance. We recommend that excess mortality be calculated using age- and sex-disaggregated data, as well as other studies on the indirect factors that may contribute to excess mortality. Standardizing the methods of analysing and reporting excess mortality would assist in contextualizing information from different sources. We also recommend the use of open-source tools such as the ACM Calculator to monitor excess mortality especially in low-resource countries, as these tools can provide standardized and timely information that may help decision-makers to optimize the use of health resources and subsequently contribute to the achievement of Sustainable Development Goals in strengthening the capacity of developing countries for early warning, risk reduction and management of national and global health risks.
